# Synaptic Information Transmission in a Two-State Model of Short-Term Facilitation

**DOI:** 10.3390/e21080756

**Published:** 2019-08-02

**Authors:** Mehrdad Salmasi, Martin Stemmler, Stefan Glasauer, Alex Loebel

**Affiliations:** 1 Graduate School of Systemic Neurosciences, Ludwig-Maximilians-Universität München, 82152 Planegg-Martinsried, Germany; 2 Bernstein Center for Computational Neuroscience Munich, 82152 Planegg-Martinsried, Germany; 3 German Center for Vertigo and Balance Disorders, Ludwig-Maximilians-Universität, 81377 Munich, Germany; 4 Department of Biology II, Ludwig-Maximilians-Universität München, 82152 Planegg-Martinsried, Germany; 5 Computational Neuroscience, Brandenburg University of Technology Cottbus-Senftenberg, 03046 Cottbus, Germany

**Keywords:** short-term synaptic facilitation, release site, information theory, binary asymmetric channel, mutual information rate, information bound

## Abstract

Action potentials (spikes) can trigger the release of a neurotransmitter at chemical synapses between neurons. Such release is uncertain, as it occurs only with a certain probability. Moreover, synaptic release can occur independently of an action potential (asynchronous release) and depends on the history of synaptic activity. We focus here on short-term synaptic facilitation, in which a sequence of action potentials can temporarily increase the release probability of the synapse. In contrast to the phenomenon of short-term depression, quantifying the information transmission in facilitating synapses remains to be done. We find rigorous lower and upper bounds for the rate of information transmission in a model of synaptic facilitation. We treat the synapse as a two-state binary asymmetric channel, in which the arrival of an action potential shifts the synapse to a facilitated state, while in the absence of a spike, the synapse returns to its baseline state. The information bounds are functions of both the asynchronous and synchronous release parameters. If synchronous release facilitates more than asynchronous release, the mutual information rate increases. In contrast, short-term facilitation degrades information transmission when the synchronous release probability is intrinsically high. As synaptic release is energetically expensive, we exploit the information bounds to determine the energy–information trade-off in facilitating synapses. We show that unlike information rate, the energy-normalized information rate is robust with respect to variations in the strength of facilitation.

## 1. Introduction

Action potentials are the key carriers of information in the brain. The arrival of an action potential at a synapse opens calcium channels in the presynaptic site, which leads to the release of vesicles filled with neurotransmitters [1]. In turn, the released neurotransmitters activate post-synaptic receptors, thereby leading to a change in the post-synaptic potential.

This process of release, however, is stochastic. The release probability is affected by the level of intracellular calcium and the size of the readily releasable pool of vesicles [2,3]. Moreover, the release of a vesicle is not necessarily synchronized with the spiking process; a synapse may release asynchronously tens of milliseconds after the arrival of an action potential [4], or sometimes even spontaneously [5].

The release properties of a synapse also change on different time scales. The successive release of vesicles can deplete the pool of vesicles, thereby depressing the synapse. On the other hand, a sequence of action potentials with short inter-spike intervals can “prime” the release mechanism and increase the release probability, inducing short-term facilitation [6].

Several studies have addressed the modulatory role of short-term depression on synaptic information transmission [7,8,9]. In contrast, the information rate of a facilitating synapse is not yet fully understood, though it has been suggested that short-term facilitation temporally filters the incoming spike train [10].

To study the impact of short-term facilitation on synaptic information efficacy, we employ a binary asymmetric channel with two states. The model synapse switches between a baseline state and facilitated state based on the history of the input. Each state has distinct release probabilities, both for synchronous and asynchronous release. We derive a lower bound and an upper bound for the mutual information rate of such a facilitating synapse and assess the functional role of short-term facilitation on the synaptic information efficacy.

Short-term facilitation increases the release probability and consequently raises the metabolic energy consumption of the synapse [11]. We calculate the rate of information transmission per unit of energy to evaluate the compromises that a facilitating synapse makes to balance energy consumption and information transmission.

## 2. Synapse Model and Information Bounds

**Figure 1:**
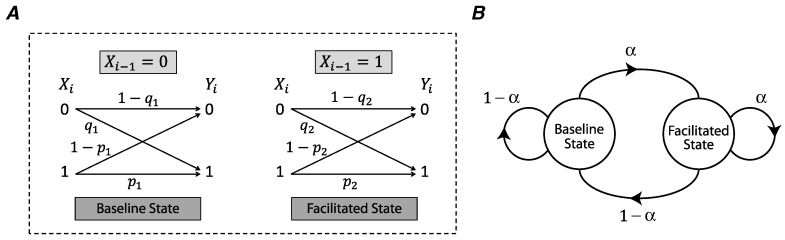
(**A**) Short-term facilitation in a synapse is modeled by a binary asymmetric channel whose state at time *i* is determined by the previous input, $X_{i-1}$. If $X_{i-1}=0$, the synapse remains in the baseline state; the synapse goes to the facilitated state after an action potential, $X_{i-1}=1$. (**B**) The transition between the baseline state and the facilitated state is modeled by a two-state Markov chain and the transition probabilities are determined by the normalized input spike rate α.

We use a binary asymmetric channel to model the stochasticity of release in a synapse (Figure 1A) [12]. The input of the model is the presynaptic spike process $X={\left\{X_{i}\right\}}_{i=0}^{\infty }$, where $X_{i}$ is a binary random variable corresponding to the presence ($X_{i}=1$) or absence ($X_{i}=0$) of a spike at time *i*. We assume that *X* is an i.i.d. random process, and $X_{i}$ is a Bernoulli random variable with $P(X_{i}=1)=\alpha$. The output process of the channel $Y={\left\{Y_{i}\right\}}_{i=0}^{\infty }$, represents a release ($Y_{i}=1$) or lack of release ($Y_{i}=0$) at time *i*. The synchronous spike-evoked release probability is characterized as $P(Y_{i}=1|X_{i}=1)$, and asynchronous release probability as $P(Y_{i}=1|X_{i}=0)$.

In short-term synaptic facilitation, a presynaptic input spike facilitates the synaptic release for the next spike. We model this phenomenon as a binary asymmetric channel whose state is determined by the previous input of the channel (Figure 1A). In the absence of a presynaptic spike ($X_{i-1}=0$), the channel is in the baseline state and the probabilities of synchronous spike-evoked and asynchronous release are $p_{1}$ and $q_{1}$. If a presynaptic spike occurs at time i−1, i.e., $X_{i-1}=1$, the state of the channel is switched to the facilitated state and the synchronous and asynchronous release probabilities are increased to $p_{2}$ and $q_{2}$ as follows,

(1) $$p_{2}=u\left(p_{max}-p_{1}\right)+p_{1},$$

(2) $$q_{2}=v\left(q_{max}-q_{1}\right)+q_{1}.$$

Here, *u* and *v* are facilitation coefficients of synchronous and asynchronous release probabilities (0≤u,v≤1), and $p_{max}$ and $q_{max}$ are the maximum release probabilities of these two modes of release. A Markov chain describes the transitions between the baseline state and the facilitated state, and the transition probabilities correspond to the presence or absence of an action potential in the presynaptic neuron (Figure 1B).

If $R_{1}$ and $R_{2}$ represent the mutual information rates of the binary asymmetric channels corresponding to the baseline state and facilitated state, then for i∈{1,2},

(3) $$R_{i}=h\left(\alpha p_{i}+\bar{\alpha }q_{i}\right)-\alpha h\left(p_{i}\right)-\bar{\alpha }h\left(q_{i}\right),$$

where $\bar{x}≜1-x$ and $h\left(x\right)=-x\log_{2}\left(x\right)-\bar{x}\log_{2}\left(\bar{x}\right)$. First we derive a lower bound for the information rate between the input spike process *X* and the output process of the release site *Y* (the proofs for the theorems are in the Appendix A).

**Theorem** **1** (Lower Bound)**.** *Let $R_{F}$ denote the mutual information rate of a synapse with short-term facilitation, modeled by the two-state binary asymmetric channel (Figure 1A). Then $R_{\mathrm{LB}}=\bar{\alpha }R_{1}+\alpha R_{2}$ is a lower bound for $R_{F}$.*

Since $\alpha =P(X_{i}=1)$, $R_{\mathrm{LB}}$ is the statistical average over the mutual information rates of the two constituent states of the release site. Therefore, our theorem shows that at least in this simple model of facilitation, the mutual information rate is higher than the statistical average over the mutual information rates of the single states. This contrasts with the result for the two-state model of depression [12], for which $\bar{\alpha }R_{1}+\alpha R_{2}$ is an exact result for the mutual information rate.

**Theorem** **2** (Lower Bound)**.** *The mutual information rate of the two-state model of facilitation is upper-bounded by*

(4) $$R_{\mathrm{UB}}=u_{7}-u_{6},$$

*where*

(5) $$u_{1}=\bar{\alpha }\left(\alpha p_{1}+\bar{\alpha }q_{1}\right),$$

(6) $$u_{2}=\bar{\alpha }q_{1}+\alpha q_{2},$$

(7) $$u_{3}=\alpha \left(\alpha p_{2}+\bar{\alpha }q_{2}\right),$$

(8) $$u_{4}=\bar{\alpha }p_{1}+\alpha p_{2},$$

(9) $$u_{5}=u_{1}+u_{3},$$

(10) $$u_{6}=h\left(q_{1}\right){\bar{\alpha }}^{2}+\left(h\left(p_{1}\right)+h\left(q_{2}\right)\right)\alpha \bar{\alpha }+h\left(p_{2}\right)\alpha^{2},$$

(11) $$u_{7}=h\left(\frac{u_{1}u_{2}+u_{3}u_{4}}{u_{5}}\right)u_{5}+h\left(\frac{u_{1}\bar{u_{2}}+u_{3}\bar{u_{4}}}{\bar{u_{5}}}\right)\bar{u_{5}}.$$

In a facilitating synapse, the release probability and consequently, the energy consumption of the synapse increases. We define the ratio of the mutual information rate by the release probability as the energy-normalized information rate of the synapse. The energy-normalized information rate of the synapse without facilitation, denoted by $R_{1}^{\left(E\right)}$, is then

(12) $$R_{1}^{\left(E\right)}=R_{1}/\left(\alpha p_{1}+\bar{\alpha }q_{1}\right).$$

Moreover, the release probability of the synapse in the two-state model of facilitation is

(13) $$P\left(Y_{i}=1\right)=\bar{\alpha }\left(\alpha p_{1}+\bar{\alpha }q_{1}\right)+\alpha \left(\alpha p_{2}+\bar{\alpha }q_{2}\right),$$

which is independent of *i*. Hence, the energy-normalized information rate of a facilitated synapse, $R_{F}^{\left(E\right)}$, as well as the lower and upper bounds of energy-normalized information rate, $R_{\mathrm{LB}}^{\left(E\right)}$ and $R_{\mathrm{UB}}^{\left(E\right)}$, are calculated by dividing $R_{F}$, $R_{\mathrm{LB}}$, and $R_{\mathrm{UB}}$ by $\bar{\alpha }\left(\alpha p_{1}+\bar{\alpha }q_{1}\right)+\alpha \left(\alpha p_{2}+\bar{\alpha }q_{2}\right)$.

## 3. Results

**Figure 2:**
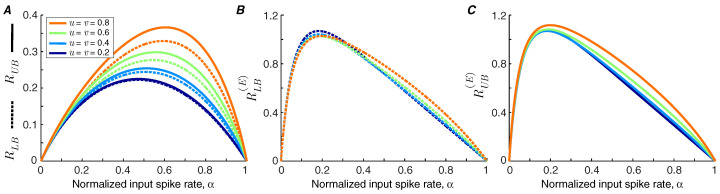
(**A**) Bounds of information rate in a synapse with short-term facilitation. The lower bound and upper bound are plotted as a function of α for different values of facilitation coefficients, *u* and *v*. (**B**) The lower bound of energy-normalized information rate of a synapse under short-term facilitation. (**C**) The upper bound of energy-normalized information rate. The model parameters are $p_{1}=0.5$, $q_{1}=0.05$, $p_{max}=1$, and $q_{max}=0.2$.

We use Theorems 1 and 2 to calculate the lower bound and upper bound of the information transmission rate of a synapse under short-term facilitation (Figure 2A). It is shown that the bounds are tighter for synapses with lower facilitation levels. We find that the information rate increases with the level of facilitation. By contrast, the bounds on the energy-normalized information rate of the synapse are relatively invariant to the strength of facilitation (Figure 2B,C). This finding indicates that a synapse can change the balance between its energy consumption and transmission rate by altering its level of facilitation without affecting the information rate per release.

**Figure 3:**
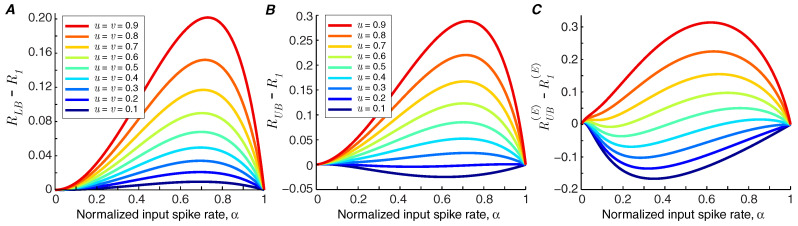
(**A**) The difference between $R_{\mathrm{LB}}$ and $R_{1}$ against input spike rate for various facilitation coefficients. It is assumed that the facilitation coefficients of synchronous spike-evoked release and asynchronous release are identical, u=v. (**B**) The difference between $R_{\mathrm{UB}}$ and $R_{1}$. (**C**) The difference between $R_{\mathrm{UB}}^{\left(E\right)}$ and $R_{1}^{\left(E\right)}$. In (**B**) and (**C**), the facilitation coefficient of asynchronous release is fixed at v=0.5. The other parameters are $p_{1}=0.5$, $q_{1}=0.05$, $p_{max}=1$ and $q_{max}=0.2$.

If the lower bound of information rate, $R_{\mathrm{LB}}$, is greater than the information rate of the synapse in the baseline state, $R_{1}$, we can conclude that short-term facilitation increases the mutual information rate of the synapse (i.e., $R_{F}>R_{1}$). In Figure 3A, it is shown that for the modeled synapse (with $p_{1}=0.5$ and $q_{1}=0.05$), short-term facilitation always increases the mutual information rate, provided that synchronous spike-evoked release and asynchronous release are facilitated equally, i.e., u=v.

Recent findings suggest that synchronous and asynchronous release may be governed by different mechanisms [4], and consequently they may show distinct levels of facilitation. We study the impact of different facilitation coefficients in the modeled synapse by fixing the facilitation coefficient of asynchronous release at v=0.5 and calculate the information bounds for different values of *u*. Short-term facilitation reduces the mutual information rate of the synapse if the upper bound of the rate of the synapse, $R_{\mathrm{UB}}$, goes below the information rate of the synapse without facilitation, $R_{1}$. Figure 3B shows that short-term facilitation degrades the information rate of the synapse if the facilitation level of synchronous release is much lower than that of asynchronous release. The degrading effect of facilitation is pronounced when we compare the upper bound of the energy-normalized information rate of the synapse with facilitation, $R_{\mathrm{UB}}^{\left(E\right)}$, with the energy-normalized information rate of a static synapse, $R_{1}^{\left(E\right)}$. The values below zero in Figure 3C show the operating points of synapses in which facilitation reduces the energy-normalized information rate.

**Figure 4:**
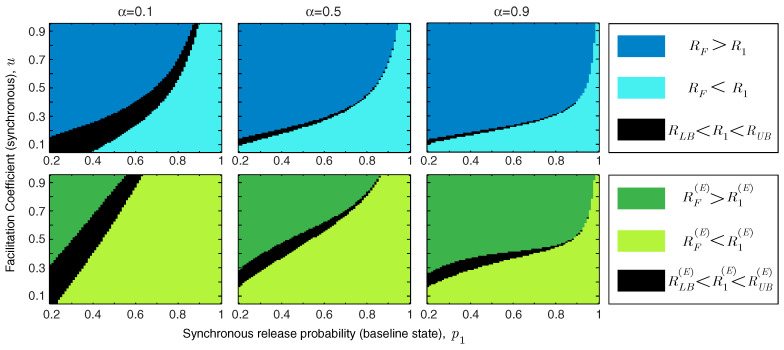
The regime of parameters (*u* and $p_{1}$) for which short-term facilitation increases/decreases the mutual information rate or energy-normalized information rate of the synapse. Asynchronous release is fixed at $q_{1}=0.05$ and v=0.5. The other parameters are $p_{max}=1$ and $q_{max}=0.2$.

In addition to the facilitation coefficient, the release probability of the synapse in the baseline state plays a critical role in determining the functional role of short-term facilitation. We study the interaction between *u* and $p_{1}$ in Figure 4. We show the regime of parameters for which short-term facilitation increases/decreases the mutual information rate and energy-normalized information rate of the synapse. If $R_{\mathrm{LB}}<R_{1}<R_{\mathrm{UB}}$ (or $R_{\mathrm{LB}}^{\left(E\right)}<R_{1}^{\left(E\right)}<R_{\mathrm{UB}}^{\left(E\right)}$), the bounds cannot specify whether facilitation increases or decreases the rate of information transmission (or energy-normalized information rate); these regions are marked in black in Figure 4. We show that for an unreliable synapse (with small $p_{1}$) and relatively large facilitation coefficient, *u*, short-term facilitation increases both mutual information rate and energy-normalized information rate of the synapse, since the enhancement of synchronous release dominates the rise of asynchronous release. Interestingly, it has been observed that for many facilitating synapses the baseline release probability is quite low [13,14]. For synapses that are more reliable *a priori*, the relative facilitation of asynchronous releases counteracts the improvement in the information rate. In reliable synapses (with higher values of $p_{1}$) and relatively small facilitation coefficients, short-term facilitation not only decreases the energy-normalized information rate of the synapse but also drops the information transmission rate. In addition, Figure 4 shows that higher input spike rates expand the range of synaptic parameters ($p_{1}$ and *u*) for which short-term facilitation enhances the rate-energy performance of the synapse.

**Figure 5:**
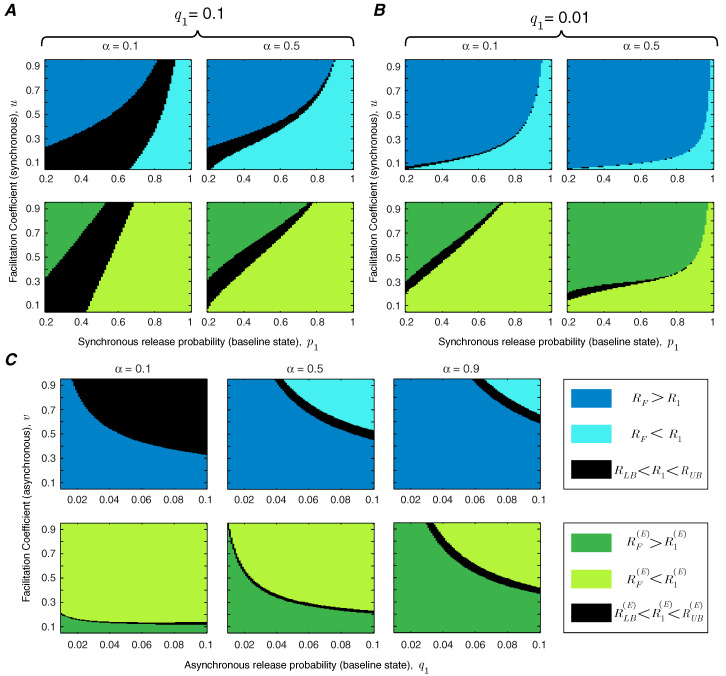
The functional impact of asynchronous release. (**A**) The range of synchronous release parameters, *u* and $p_{1}$, in which short-term facilitation enhances energy-rate efficiency of the synapse; the asynchronous release probability is $q_{1}=0.1$. (**B**) Similar to (**A**) for $q_{1}=0.01$. (**C**) The functional classes of short-term facilitation are modified by the baseline release probability, $q_{1}$, and facilitation coefficient, *v*, of the asynchronous release probability. The other simulation parameters are v=0.5 in (**A**,**B**), and $p_{1}=0.5$ and u=0.5 in (**C**). For all simulations, $p_{max}=1$ and $q_{max}=4q_{1}$.

To study the effect of asynchronous release, we repeat the analysis of Figure 4 for very high ($q_{1}\;=\;0.1$) and very low ($q_{1}=0.01$) asynchronous release probabilities. Comparing Figure 5A,B reveals that decreasing the level of asynchronous release expands the range of synchronous release parameters, *u* and $p_{1}$, for which short-term facilitation increases the mutual information rate and energy-normalized information rate. We also study the interaction between asynchronous release probability $q_{1}$ and the facilitation coefficient of asynchronous release *v*, keeping the parameters of synchronous release fixed at $p_{1}=0.5$ and u=0.5 (Figure 5C). To benefit from short-term facilitation, the synapse needs to decrease the release probability and/or the facilitation coefficient of the asynchronous mode of release. For synapses with very high asynchronous release probabilities, short-term facilitation can still boost the information rate and energy-normalized rate of the synapse, provided that the facilitation coefficient of the asynchronous release is small enough. Similar to the results in Figure 4, by increasing the normalized spike rate, the synapse spends more time in the facilitated state and therefore, the impact of short-term facilitation on rate-energy efficiency of the synapse is enhanced.

**Figure 6:**
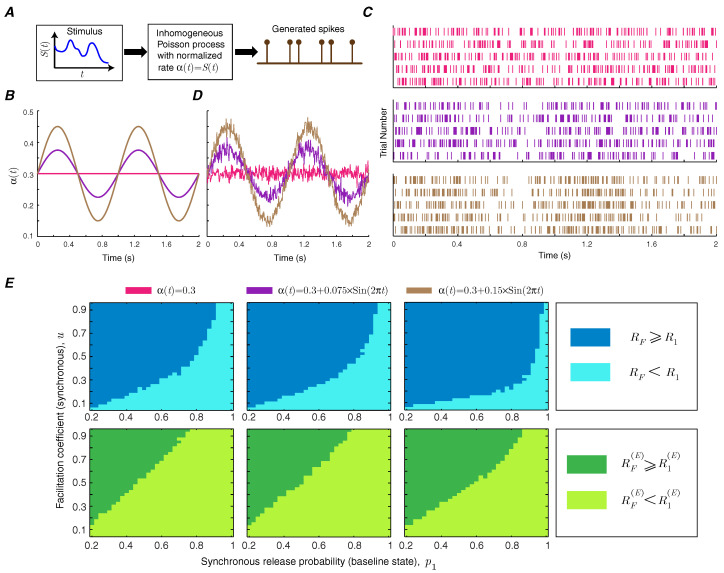
(**A**) Generation of correlated spike trains. (**B**) Sinusoidal stimulus signals, with a frequency of 1 Hz, average value of 0.3, and amplitudes 0, 0.075, and 0.15, are used as the normalized rate α(t) of the inhomogeneous Poisson process. (**C**) The spike raster plots of the simulated neurons (5 trials for each amplitude). (**D**) The estimation of the instantaneous neuronal firing rate from 1000 trials. (**E**) Functional classes of short-term facilitation for correlated input. The first column corresponds to the uncorrelated input (α(t)=0.3) and the second and third columns correspond to the correlated spike trains generated by sinusoidal stimulus signals with amplitudes 0.075 and 0.15. The other simulation parameters are $q_{1}=0.05$, v=0.5, $p_{max}=1$, and $q_{max}=0.2$.

Short-term facilitation creates a memory for the synapse, since the release probability of the synapse depends on the history of spiking activity. It is, therefore, important to study how short-term facilitation modulates information transmission rate in synapses with temporally correlated spike trains by making the Poisson rate of the input spike train time-dependent [15] (Figure 6A). We use a sinusoidal rate stimulus with a frequency of 1 Hz on top of a baseline rate (Figure 6B) and use the context-tree weighting algorithm to numerically estimate the mutual information and energy-normalized information rates of the facilitating synapse [16]. The amplitude of the sinusoidal signal specifies the level of correlation. The raster plots of the neurons show the synchrony between the spiking activity of the neuron and the sinusoidal instantaneous rate (Figure 6C). The instantaneous firing rate, averaged over 1000 trials, provides a good estimate of the stimulus (Figure 6D). The functional classes of short-term facilitation are calculated as a function of baseline release probability and facilitation coefficient of synchronous release for different levels of correlation. This numerical analysis shows that correlations in the presynaptic spike train can slightly enlarge the regions in which short-term facilitation increases the mutual information rate and energy-normalized information rate (Figure 6E).

**Figure 7:**
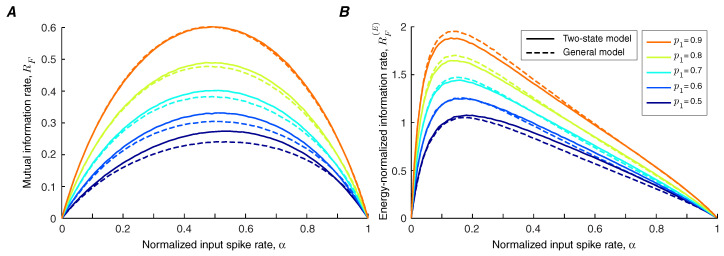
(**A**) Mutual information rate of the two-state model (solid lines) and general model (dashed lines) of short-term facilitation as a function of normalized input spike rate for various values of baseline synchronous release probability, $p_{1}$. (**B**) Similar to (**A**) for energy-normalized information rates. The simulation parameters are $q_{1}=0.05$, u=v=0.5, $p_{max}=1$, $q_{max}=0.2$, $\tau_{{}_{L,p}}=\tau_{{}_{L,q}}=250$ msec, and $\tau_{{}_{f,p}}=\tau_{{}_{f,q}}=20$ msec.

In the general model of facilitation, it is assumed that the state of the synapse at time *i* is affected not only by the spiking activity of the presynaptic neuron at time i−1, but also by the whole history of the spiking events. Synchronous and asynchronous release probabilities converge to the limit probabilities, $p^{\left(L\right)}$ and $q^{\left(L\right)}$, exponentially by time constants $\tau_{{}_{L,p}}$ and $\tau_{{}_{L,q}}$. The arrival of an action potential at time *i* increases the limit probabilities $p_{i}^{\left(L\right)}$ and $q_{i}^{\left(L\right)}$ by $u(p_{max}-p_{i}^{\left(L\right)})$ and $v(q_{max}-q_{i}^{\left(L\right)})$ respectively; the initial values of the limit probabilities are $p_{0}^{\left(L\right)}=p_{1}$ and $q_{0}^{\left(L\right)}=q_{1}$. In the quiescent intervals, the limit probabilities decay to the baseline values, $p_{1}$ and $q_{1}$, by facilitation decay time constants, $\tau_{{}_{f,p}}$ and $\tau_{{}_{f,q}}$. The numerical methods are used to compare the synaptic information efficacy of the two-state model with the general model of short-term facilitation. We show that the two-state model provides a good approximation for the mutual information rate (Figure 7A) and energy-normalized information rate (Figure 7B) of a facilitating synapse, provided that the facilitation decays rapidly. If the facilitation decay time constant is large, similar to the approach in [12], the parameters of the two-state model can be tuned to provide a better estimation.

## 4. Discussion

We studied how prior spikes, by facilitating the release of neurotransmitter at a synapse, modulate the rate of synaptic information transfer. Most components of neural hardware are noisy, hybrid analog-digital devices. In particular, the synapse maps quite naturally onto an asymmetric binary channel in communication theory. Some neurons, such as thalamic relay neurons, act as nodes in a network for long-range communication using spikes, so it is natural to quantify the performance of the synapses in bits [17,18,19,20,21,22,23]. Synaptic information efficacy quantifies the amount of information that the post-synaptic potential contains about the spiking activity of the presynaptic neuron. This analysis, however, does not guarantee that the post-synaptic neuron accesses or uses this information, which rather depends on the biophysical mechanisms of encoding and decoding.

To capture the phenomenon of facilitation, we made the binary asymmetric channel have two states. The resulting model permits the short-term dynamics of synchronous and asynchronous releases to be different, which enabled us to assess the impact of each release mode on the efficacy of synaptic information transfer. We first assumed identical facilitation coefficients for synchronous and asynchronous release (i.e., u=v) and demonstrated that the lower bound of information rate of a facilitating synapse is higher than the information rate of a static synapse (Figure 3A). We were, therefore, able to show that synapses quantifiably transmit more information through short-term facilitation, as long as synchronous and asynchronous release of neurotransmitter obey the same dynamics. Indeed, the increase in information can outweigh the higher energy consumption, as measured by the energy-normalized information rate, provided that synchronous release is facilitated more than asynchronous release. In contrast, when facilitation enhances the asynchronous component of release more strongly than the synchronous component, short-term facilitation would have the opposite effect, namely to decrease synaptic information efficacy.

In previous work, we studied the information transmission in a synapse during short-term depression [9,12]. There, the state of the binary asymmetric channel, which models the synapse, depended on the history of the output $\left(Y_{i-1},Y_{i-2},\ldots ,Y_{1}\right)$. Facilitation, in contrast, depends on the history of the input $\left(X_{i-1},X_{i-2},\ldots ,X_{1}\right)$. This simple change makes the problem much more challenging mathematically, as it is, in fact, isomorphic to an unsolved problem in information theory, namely the entropy rate of a hidden Markov process [24]. Nevertheless, the lower bound we derive for a facilitating synapse mirrors the exact result we had derived earlier for short-term depression. Moreover, in practice, this bound is fairly tight.

The bounds derived here are only a first step towards understanding information transmission in facilitating synapses. The two-state binary asymmetric channel simplifies the process of facilitation by making the release probability depend only on the presence or absence of a presynaptic spike at the previous time-point. Yet when the facilitation decays rapidly, the two-state model converges in behavior to a more general model that considers the entire history of spiking.

Our model ignores the possibility of temporal correlations in the presynaptic spike train. Instead, in line with many other studies, the time series of spikes were assumed to obey Poisson statistics [8,22,25]. This simplification made the information-theoretic analysis of the synapse tractable and helped us to derive the upper bound and lower bound of information rate. Different methods have been suggested for modeling correlated spike trains, such as inhomogeneous Poisson processes [15,26], autoregressive point processes [27], and random spike selections from a set of spike trains [15]. We used an inhomogeneous Poisson process to generate correlated spike trains and estimated the mutual information rate and energy-normalized information rate of the synapse numerically. In the future, it will be of interest to study the effect of correlated input on the information efficacy of the general model of facilitation in which the release probabilities are determined by the whole history of spiking activity.

In this study, we have assumed that in response to an incoming action potential, the release site releases at most one vesicle. To capture multiple releases, the model should be extended to a communication channel with binary input and multiple outputs. The analysis of this channel will reveal the impact of multiple releases on the mutual information rate of a static and dynamic synapse.

## Appendix A

**Proof** **of** **Theorem** **1.** Let $X^{n}≜\left(X_{1},X_{2},\ldots ,X_{n}\right)$. By definition,

(A1) $$R_{F}=\lim_{n\to \infty }\frac{1}{n}I\left(X^{n};Y^{n}\right),$$

where

(A2) $$I\left(X^{n};Y^{n}\right)=H\left(Y^{n}\right)-H\left(Y^{n}|X^{n}\right).$$

The chain rule implies that

(A3) $$H\left(Y^{n}\right)=\sum_{i=1}^{n}H(Y_{i}|Y^{i-1}),$$

(A4) $$H\left(Y^{n}|X^{n}\right)=\sum_{i=1}^{n}H(Y_{i}|Y^{i-1},X^{n}).$$

The model in Figure 1A posits that $Y_{i}$ depends only on $X_{i}$ and $X_{i-1}$. Given $X_{i-1}$, $Y_{i}$ is independent of $Y^{i-1}$ and consequently

(A5) $$H\left(Y_{i}|Y^{i-1},X^{n}\right)=H\left(Y_{i}|X_{i-1},X_{i}\right).$$

Also

(A6) $$H\left(Y_{i}|Y^{i-1},X_{i-1}\right)=H\left(Y_{i}|X_{i-1}\right).$$

From (A6),

(A7) $$\begin{array}{c} H\left(Y_{i}|Y^{i-1}\right)-H\left(Y_{i}|X_{i-1}\right)=I\left(Y_{i};X_{i-1}|Y^{i-1}\right)\geq 0. \end{array}$$

Hence,

(A8) $$H\left(Y_{i}|Y^{i-1}\right)\geq H\left(Y_{i}|X_{i-1}\right),$$

and together with (A3),

(A9) $$H\left(Y^{n}\right)\geq \sum_{i=1}^{n}H(Y_{i}|X_{i-1}),$$

From (A4) and (A5),

(A10) $$H\left(Y^{n}|X^{n}\right)=\sum_{i=1}^{n}H(Y_{i}|X_{i-1},X_{i}).$$

By applying (A9) and (A10) to (A2),

(A11) $$\begin{array}{cc} I(X^{n};Y^{n}) & \geq \sum_{i=1}^{n}\left(H\left(Y_{i}|X_{i-1}\right)-H\left(Y_{i}|X_{i-1},X_{i}\right)\right) \end{array}$$

(A12) $$\begin{array}{c} =\sum_{i=1}^{n}I(Y_{i};X_{i}|X_{i-1}). \end{array}$$

Using the definition of conditional mutual information (for i≥2),

(A13) $$\begin{array}{cc} I(Y_{i};X_{i}|X_{i-1}) & =I\left(Y_{i};X_{i}|X_{i-1}=0\right)P\left(X_{i-1}=0\right)+I\left(Y_{i};X_{i}|X_{i-1}=1\right)P\left(X_{i-1}=1\right) \end{array}$$

(A14) $$\begin{array}{c} =\bar{\alpha }R_{1}+\alpha R_{2}. \end{array}$$

Finally, the lemma follows from (A1), (A12) and (A14). □

**Proof** **of** **Theorem** **2.** The non-negativity of mutual information implies that

(A15) $$H\left(Y_{i}|Y^{i-1}\right)\leq H\left(Y_{i}|Y_{i-1}\right).$$

By conditioning on $X_{i-2}$,

(A16) $$P\left(Y_{i-1}=1\right)=\left(\alpha p_{1}+\bar{\alpha }q_{1}\right)\bar{\alpha }+\left(\alpha p_{2}+\bar{\alpha }q_{2}\right)\alpha =u_{5},$$

and similarly

(A17) $$P\left(Y_{i-1}=0\right)=\left(\alpha \bar{p_{1}}+\bar{\alpha }\;\bar{q_{1}}\right)\bar{\alpha }+\left(\alpha \bar{p_{2}}+\bar{\alpha }\;\bar{q_{2}}\right)\alpha =\bar{u_{5}}.$$

Moreover

(A18) $$H\left(Y_{i}|Y_{i-1}=0\right)=h\left(P(Y_{i}=1|Y_{i-1}=0)\right),$$

(A19) $$H\left(Y_{i}|Y_{i-1}=1\right)=h\left(P(Y_{i}=1|Y_{i-1}=1)\right),$$

and

(A20) $$P\left(Y_{i}=1|Y_{i-1}=0\right)=\sum_{x_{i-1}\in \left\{0,1\right\}}P\left(Y_{i}=1|Y_{i-1}=0,X_{i-1}=x_{i-1}\right)\times P\left(X_{i-1}=x_{i-1}|Y_{i-1}=0\right).$$

We have

(A21) $$\begin{array}{cc} P(Y_{i}=1|Y_{i-1}=0,X_{i-1}=0) & =P(Y_{i}=1|X_{i-1}=0) \end{array}$$

(A22) $$\begin{array}{c} =\alpha p_{1}+\bar{\alpha }q_{1}, \end{array}$$

(A23) $$\begin{array}{cc} P(Y_{i}=1|Y_{i-1}=0,X_{i-1}=1) & =P(Y_{i}=1|X_{i-1}=1) \end{array}$$

(A24) $$\begin{array}{c} =\alpha p_{2}+\bar{\alpha }q_{2}. \end{array}$$

Also

(A25) $$P\left(X_{i-1}=1|Y_{i-1}=0\right)=\frac{P\left(Y_{i-1}=0|X_{i-1}=1\right)P\left(X_{i-1}=1\right)}{P(Y_{i-1}=0)},$$

By conditioning on $X_{i-2}$, and given the fact that $X_{i-2}$ and $X_{i-1}$ are independent,

(A26) $$\begin{array}{c} P\left(Y_{i-1}=0|X_{i-1}=1\right)=\bar{\alpha }\;\bar{p_{1}}+\alpha \bar{p_{2}}=\bar{u_{4}}. \end{array}$$

Hence,

(A27) $$P\left(X_{i-1}=1|Y_{i-1}=0\right)=\frac{\alpha \bar{u_{4}}}{\bar{u_{5}}},$$

and similarly, we can show that

(A28) $$P\left(X_{i-1}=0|Y_{i-1}=0\right)=\frac{\bar{\alpha }\;\bar{u_{2}}}{\bar{u_{5}}}.$$

Therefore,

(A29) $$P\left(Y_{i}=1|Y_{i-1}=0\right)=\frac{u_{1}\bar{u_{2}}+u_{3}\bar{u_{4}}}{\bar{u_{5}}}.$$

With the same approach,

(A30) $$P\left(Y_{i}=1|Y_{i-1}=1\right)=\frac{u_{1}u_{2}+u_{3}u_{4}}{u_{5}}.$$

The conditional entropy $H(Y_{i}|Y_{i-1})$ can be written as

(A31) $$H\left(Y_{i}|Y_{i-1}\right)=H\left(Y_{i}|Y_{i-1}=1\right)P\left(Y_{i-1}=1\right)+H\left(Y_{i}|Y_{i-1}=0\right)P\left(Y_{i-1}=0\right),$$

and for i>2, we can infer from (A16)–(A19), (A29), and (A30),

(A32) $$\begin{array}{cc} H(Y_{i}|Y_{i-1}) & =h\left(\frac{u_{1}u_{2}+u_{3}u_{4}}{u_{5}}\right)u_{5}+h\left(\frac{u_{1}\bar{u_{2}}+u_{3}\bar{u_{4}}}{\bar{u_{5}}}\right)\bar{u_{5}} \end{array}$$

(A33) $$\begin{array}{c} =u_{7}, \end{array}$$

We also conclude that $H(Y_{i}|Y_{i-1})$ is independent of *i* for i>2. Moreover, we can easily obtain

(A34) $$\begin{array}{cc} H(Y_{i}|X_{i-1},X_{i}) & =h\left(q_{1}\right){\bar{\alpha }}^{2}+\left(h\left(p_{1}\right)+h\left(q_{2}\right)\right)\alpha \bar{\alpha }+h\left(p_{2}\right)\alpha^{2} \end{array}$$

(A35) $$\begin{array}{c} =u_{6}. \end{array}$$

From (A5) and (A15),

(A36) $$\begin{array}{cc} I(X^{n};Y^{n}) & =\sum_{i=1}^{n}\left(H\left(Y_{i}|Y^{i-1}\right)-H\left(Y_{i}|X_{i},X_{i-1}\right)\right) \end{array}$$

(A37) $$\begin{array}{c} \leq \sum_{i=1}^{n}\left(H\left(Y_{i}|Y_{i-1}\right)-H\left(Y_{i}|X_{i},X_{i-1}\right)\right). \end{array}$$

Therefore, from (A33) and (A35),

(A38) $$I\left(X^{n};Y^{n}\right)\leq I\left(Y_{1};X_{1}\right)+H\left(Y_{2}|Y_{1}\right)-H\left(Y_{2}|X_{2},X_{1}\right)+\left(n-2\right)\left(u_{7}-u_{6}\right),$$

and by dividing by *n* and calculating the limit when *n* goes to infinity,

(A39) $$\begin{array}{cc} R_{F} & \leq u_{7}-u_{6}, \end{array}$$

and the proof is complete. □
